# Gogo Receptor Contributes to Retinotopic Map Formation and Prevents R1-6 Photoreceptor Axon Bundling

**DOI:** 10.1371/journal.pone.0066868

**Published:** 2013-06-24

**Authors:** Irina Hein, Takashi Suzuki, Ilona C. Grunwald Kadow

**Affiliations:** 1 Max-Planck Institute of Neurobiology, Martinsried, Germany; 2 Tokyo Institute of Technology, Nagatsuta, Midoriku, Yokohama, Japan; National Institutes of Health (NIH), United States of America

## Abstract

**Background:**

Topographic maps form the basis of neural processing in sensory systems of both vertebrate and invertebrate species. In the Drosophila visual system, neighboring R1–R6 photoreceptor axons innervate adjacent positions in the first optic ganglion, the lamina, and thereby represent visual space as a continuous map in the brain. The mechanisms responsible for the establishment of retinotopic maps remain incompletely understood.

**Results:**

Here, we show that the receptor Golden goal (Gogo) is required for R axon lamina targeting and cartridge elongation in a partially redundant fashion with local guidance cues provided by neighboring axons. Loss of function of Gogo in large clones of R axons results in aberrant R1–R6 fascicle spacing. Gogo affects target cartridge selection only indirectly as a consequence of the disordered lamina map. Interestingly, small clones of *gogo* deficient R axons perfectly integrate into a proper retinotopic map suggesting that surrounding R axons of the same or neighboring fascicles provide complementary spatial guidance. Using single photoreceptor type rescue, we show that Gogo expression exclusively in R8 cells is sufficient to mediate targeting of all photoreceptor types in the lamina. Upon lamina targeting and cartridge selection, R axons elongate within their individual cartridges. Interestingly, here Gogo prevents bundling of extending R1-6 axons.

**Conclusion:**

Taken together, we propose that Gogo contributes to retinotopic map formation in the Drosophila lamina by controlling the distribution of R1–R6 axon fascicles. In a later developmental step, the regular position of R1–R6 axons along the lamina plexus is crucial for target cartridge selection. During cartridge elongation, Gogo allows R1–R6 axons to extend centrally in the lamina cartridge.

## Introduction

Precise wiring of the visual system enables animals to perceive and respond to their visual world. In Drosophila, axons project in a topographic fashion, such that adjacent photoreceptor (R) cells connect to adjacent postsynaptic neurons [1], [2]. Thus, the retina creates a two-dimensional image of the visual environment in the brain, which is referred to as retinotopic map. Many studies in vertebrates and invertebrates revealed molecular mechanisms controlling precise columnar and layer-specific axon targeting during visual system development [3], [4], [5], [6], [7], [8], [9], [10], [11].

In the Drosophila visual system, it is thought that targeting of R cells follows a genetically hard-wired program to form complex and stereotyped microcircuits [12]. The compound eye is built of about 800 single eyes or ommatidia, each containing 8 different types of R cells that innervate different ganglia in the brain [13], [14]. The outer R1–R6 cells target to the first optic ganglion, the lamina, while the two inner R7 and R8 cells project through the lamina to innervate different layers in the underlying medulla ganglion.

Proper connectivity of R1–R6 axons in the lamina requires extraordinary precision of synaptic specificity. Due to the eye’s curvature six R1–R6 cells from six different neighboring ommatidia share the same optical axis and converge onto the same set of postsynaptic lamina neurons, resulting in synaptic units called cartridges [2], [15]. This remarkable feature of axonal resorting is referred to as neural superposition and serves the purpose of increasing light sensitivity by enhancing the signal-to-noise ratio. Cartridge assembly happens in three distinct developmental steps. During third instar larval stages ommatidial fascicles extend towards the brain in a sequential order from posterior to anterior [15], [16]. R1–R6 cells of the same ommatidium fasciculate and terminate topographically, displaying a highly ordered pattern, the initial topographic map. In the second developmental step during midpupal development, R1–R6 axons defasciculate simultaneously and extend laterally across the lamina plexus. Here, their projection pattern is invariant and directly related to the position of their cell-bodies [16]. In the last step during the second half of pupal development, R1–R6 axons turn again, elongate proximally and form synapses [14].

A previous study revealed that expression of the cadherin related surface protein Flamingo (Fmi) in the first outgrowing R8 axons appears sufficient to rescue the formation of the initial topographic map [6]. In addition, Fmi mediates correct target cartridge selection among afferents via homophilic repulsive non-cell autonomous interactions between R axons [17].

Here, we used genetic manipulation in Drosophila R axons to identify the mechanisms required for the formation of topographic visual maps. We found that the lamina map forms by using a combination of mechanisms that directly and indirectly depend on the transmembrane receptor Golden goal (Gogo), previously described as mediating R8 targeting in the medulla [18], [19]. Gogo directly mediates initial topographic map formation by guiding pioneer R8 axons to their proper targets. This is also sufficient to allow all other R axons to find their target cartridges in a Gogo-independent manner. Interestingly, we provide data comparing small and large *gogo* mutant clones suggesting that Gogo function can be compensated for by proper targeting of a sufficient number of adjacent wild-type fascicles. Additionally, Gogo is required for R axons to extend within their appropriate columns during cartridge elongation independent from its early function. Thus, our data provides initial evidence for axon repulsion not only during early targeting steps but also during R axon elongation in already assembled cartridges.

## Materials and Methods

### Fly Strains and Genetics

Flies were kept on standard Drosophila medium at 25°C. For staging, white pupae (0–1 h APF) were collected and raised for 30 and 42 hr, respectively on Drosophila standard medium at 25°C. Single cell MARCM experiments for R1–R6 axons were performed using a heat shock FLP recombinase (*hsflp*). 3^rd^ instar larvae were heat shocked for 1 h at 38°C and neurons were labeled by expressing *UAS-mCD8GFP* under the control of the pan-neuronal driver *elav-Gal4*. The following fly strains were used: *FRT80B gogo^H1675^; FRT42B fmi^E59^* (Bloomington stock center), *eyflp2* (referred to as *eyflp*) [20], *ey3.5flp; FRT80B, FRT80B 3L cl, Gmr-mCD8-mKO-myc* (monomeric Kusabira Orange; [18]), *hsflp*; 109-68-*Gal4* [21], *elav*-Gal4, *mδ*-Gal4, *tub-Gal80*, *UAS-mCD8GFP* and *UAS-gogo*.

Detailed genotypes:

eyflp;gogo experiments in larval and pupal stages: ey3.5flp/+ (or ey3.5flp/ey3.5flp); mδ-Gal4, UASmCD8-GFP/+; FRT80B, gogo^H1675^, Gmr-KO/FRT80B, tub-Gal80, 3L cl. Control: ey3.5flp/+ (or ey3.5flp/ey3.5flp; mδ-Gal4, UASmCD8-GFP/+; FRT80B, Gmr-KO/FRT80B, tub-Gal80, 3L cl. complementary MARCM genotypes: elav-Gal4, hsflp, UAS-mCD8-GFP/+;; FRT80B, gogo^H1675^/FRT80B, tub-Gal80, Gmr-mCD8-mKO-myc. reverse MARCM genotypes: elav-Gal4, hsflp, UAS-mCD8-GFP/+;; FRT80B, tub-Gal80, gogo^H1675^/FRT80B, Gmr-mCD8-mKO-myc. R8 rescue experiments: eyflp2/eyflp2; 109-68-Gal4/UAS-gogo; FRT80B, gogo^H1675^/FRT80B, 3L cl. control: eyflp2/eyflp2; 109-68-Gal4/+; FRT80B, gogo^H1675^/FRT80B, 3L cl. R4 rescue experiments: eyflp2/eyflp2; mδ-Gal4/UAS-gogo; FRT80B, gogo^H1675^/FRT80B, 3L cl. control: eyflp2/eyflp2; mδ-Gal4/+; FRT80B, gogo^H1675^/FRT80B, 3L cl. Overexpression experiments: Rh1-τlacZ/+; mδ-Gal4, UASmCD8-GFP/UAS-gogo; UAS-gogo/+. Control: Rh1-τlacZ/+; mδ-Gal4, UASmCD8-GFP/+. eyflp;gogo and eyflp;fmi experiments in adult brains: eyflp2/Rh1-τlacZ;;Gogo^H1675^, FRT80B/FRT80B. eyflp2/Rh1-τlacZ; fmi^E59^, FRT42B/FRT42B.

For MARCM experiments 3^rd^ instar larvae were heat-shocked for 1 hr at 38°C and shifted to 25°C for 42 hr.

### Immunohistochemistry and Imaging

Brains were fixed and stained as described previously [22]. Agarose sectioning of adult fly heads was performed as described in [23]. For primary antibodies we used mouse monoclonal 24B10 (1∶25 dilution, Developmental Studies Hybridoma Bank [DSHB]), rabbit monoclonal to GFP conjugated with Alexa488 (1∶200, Molecular probes), rabbit polyclonal to myc (1∶200, Gramsch),mouse monoclonal to c-myc (9E10) conjugated with TRITC (1∶200, Santa Cruz Biotechnology), rat monoclonal to elav (1∶100, DSHB) and mouse monoclonal to β-galactosidase (1∶300, Promega). Alexa Fluor-conjugated secondary antibodies (488, 568, 633; Molecular Probes) were used at 1∶250. Images were taken on an Olympus FV-1000 confocal microscope and processed in Photoshop and Illustrator (Adobe). Voronoi and DeLaunay diagrams were generated in Fijii using Delaunay/Voronoi plugin [24].

### Quantification of R4 Orientation Vectors

Clones within the lamina plexus were identified by the lack of Gmr-KO expression and gogo mutant R4 axons were identified by mCD8-GFP expression. The phenotype was quantified by measuring the orientation vectors of mutant R4 axons (minimum 10 R4 axons per lamina) with respect to the eyes equator. We calculated the equality of variances of mutant and wild-type R4 orientation vectors using Levene’s test.

### Statistical Analysis

Statistical significance for two-tailed Student’s t-test and chi test was assessed in Excel. Statistical significance for Kolmogorov–Smirnov test and Levene’s test was assessed in Python using custom written scripts from SciPy [25], [26].

## Results

### Gogo is Required for Spatial Distribution of R Cell Fascicles along the Lamina Plexus

**Figure 1:**
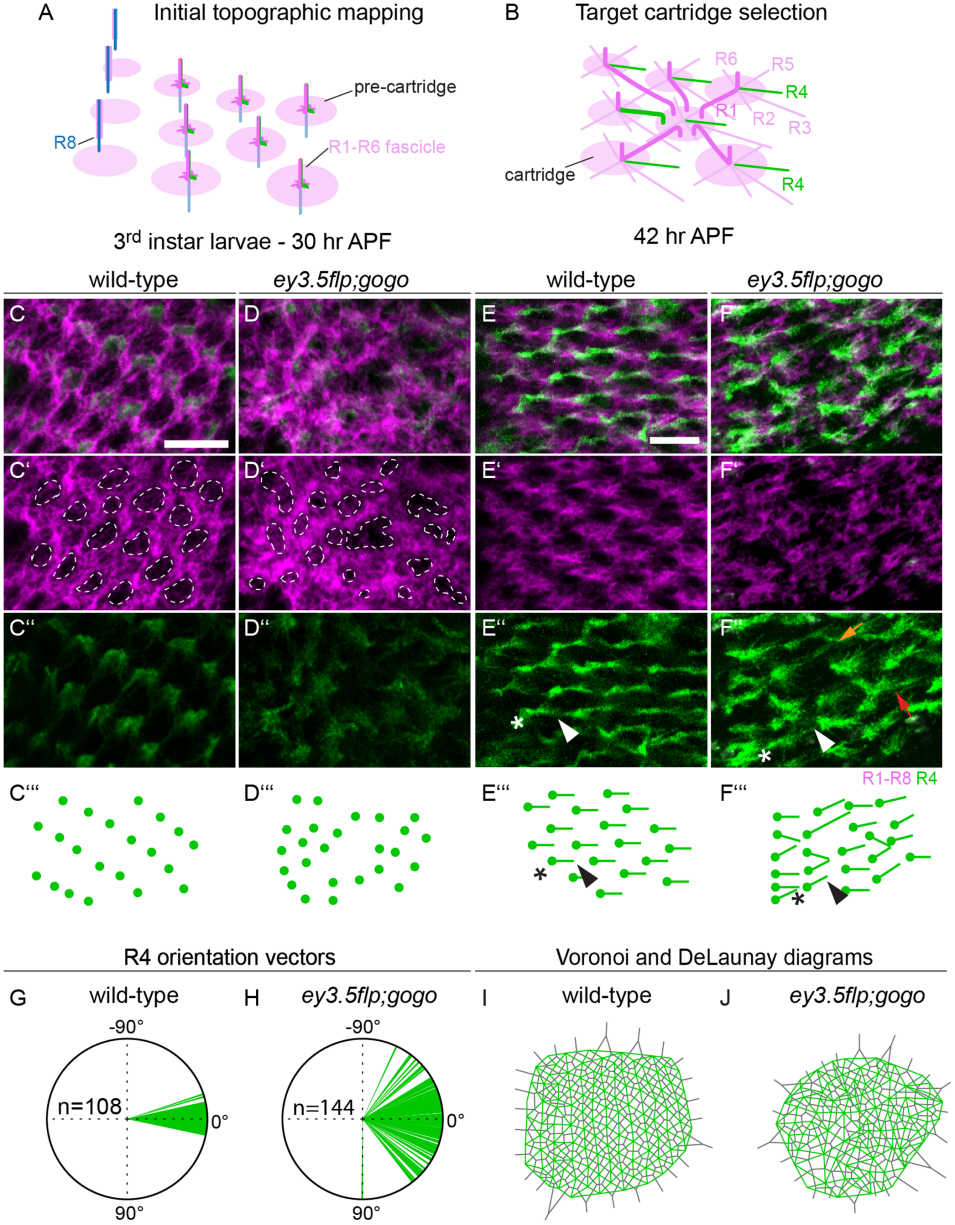
Retinotopic map formation and target cartridge selection is disrupted in the absence of Gogo. (A–B) Schematics of cartridge assembly. (A) Within each fascicle, the R8 axon extends first to the lamina plexus during larval development, followed by a sequential outgrowth of R1–R7. R1-6 axon fascicles reach the lamina plexus in a precise spatial pattern, forming the initial topographic map (30 hr APF), whereas R8 projects through the lamina to innervate the medulla. (B) Subsequently, R1-6 fascicles separate and project to different specific cartridges. Six R1–R6 axons from six different ommatidia in turn converge with one set of lamina neurons (not shown) to a single target cartridge. (C–D’’’) Confocal images and schematics of retinotopic mapping of R1–R6 fascicles in wild-type and *eyflp;gogo* background at 30 hr APF. (C–C’’’) In wild-type FRT80 controls R1–R6 fascicles terminate at the lamina plexus maintaining their equal spacing and the spatial order of their ommatidia. (D–D’’’) In *gogo* mutant background, R1–R6 fascicles fail to arrange in the correct order to neighboring axons. (E–F’’’) Sections of midpupal lamina at the onset of target cartridge selection at 42 hr APF in control and in *gogo* mutant background. Arrowheads (E–E’’’, F–F’’’) and dots (E’’’, F’’’) mark the start points and asterisks (E–E’’’, F–F’’’) the end of R4 extensions. (E–E’’’) In control animals, R4 projection pattern (green) is uniform in direction and length and the overall pattern (magenta) displays orderly distributed and uniformly sized cartridges. (F–F’’’) When Gogo is removed from the majority of R cells R4 extensions (green) are not parallel and the overall pattern of the lamina (magenta) is highly disrupted. R4 cells sometimes form long growth cones (orange arrow) or two axons converge to a single target (red arrow). (G, J) Polar plots visualize orientation vectors of R4 axons in wild-type control and mutant. (H, I) DeLaunay (green strokes) and Voronoi diagrams (grey strokes) display the uniform and irregular retinotopic mapping in wild-type (E–E’’’) and mutant (F–F’’’), respectively. Scale bars: 10 µm.

In order to identify the molecular rules that guide proper cartridge formation in the lamina, we analyzed candidate gene mutants with phenotypes in the visual system. We have previously shown that the transmembrane protein Golden goal (Gogo) is required specifically in R axons for correct cartridge assembly in the adult fly medulla [19]: Analysis of *gogo* mutant R cells at the level of lamina cartridges revealed strong hypo- and hyperinnervation defects (*eyflp;;FRT80, gogo^H1675^/FRT80, 3L cl*). We first examined the specific requirement of Gogo in R cells during different developmental time windows. To this aim, we generated genetic mosaic eyes using the FLP/FRT system [20] and expressed the FLP recombinase under the control of the R cell specific *eyeless* promoter fragment ‘ey3.5′ [9]. In these flies, the majority of R cells but not the target cells are *gogo* mutant (*ey3.5flp; mδ-Gal4, UASmCD8-GFP/+; FRT80B, gogo^H1675^, Gmr-KO/FRT80B, tub-Gal80, 3L cl*; eyflp experiments are from now on referred to as *eyflp;gogo and ey3.5flp;gogo*, respectively; for detailed genotypes see material and methods). In all experiments we used the *gogo* null allele *gogo* ^H1675^ [18]. We visualized R1–R8 axons by 24B10 antibody staining; in addition all R4 axons were labeled with mCD8-GFP using the specific promoter *mδ-Gal4* [27]. The *mδ-Gal4* drives expression in Drosophila larval stages in R3, R4 and R7, whereas in pupal stages expression was visible only in R4 and glia cells. Once R axon fascicles reach the lamina plexus, they terminate topographically with fixed relative positions to adjacent fascicles [14] that we refer to as pre-cartridges (Figure 1A). To examine retinotopic mapping, we analyzed pupae at 30 hr after puparium formation (APF) before ommatidial bundles defasciculate to spread laterally along the lamina surface selecting target cartridges. In wild-type pupae, pre-cartridges are uniformly sized and distributed in a stereotypic hexagonal array (Figure 1C–C’’’). In contrast, in the mosaic eyes, the overall lamina structure showed a disorganized pattern, and the distance between and the size of the pre-cartridges appeared irregular and variable (Figure 1D–D’’’). Thus, Gogo function in R cells is required for the orderly distribution of R cells along the lamina plexus.

We next examined R1–R6 projection patterns on the lamina surface at the onset of target cartridge selection at 42 hr AFP. The projection of each R cell subtype is characteristic in direction and length, and as a result homologous R cell subtypes display a parallel projection pattern (Figure 1B) [14], [28]. We analyzed the projection pattern of R4 during cartridge selection using a driver expressing exclusively in R4 axons at this stage (*mδ-Gal4*). As expected, in the control situation, R4 axonal extensions are uniform in direction and length (Figure 1E–E’’’). In contrast, lack of Gogo in R cell clones strongly disrupted the regularity of R4 projections (Figure 1F–F’’’): Orientation vectors for mutant R4 cells (n = 144) demonstrated that axons failed to project in parallel and consequently their distribution angle was significantly higher compared to wild-type controls (n = 108, Figure 1G–H, two-sampled Kolmogorov–Smirnov (K-S) test p<0.0005). Additionally, unlike in the wild-type control, R4 extensions differ significantly in length: While the mean length was not significantly different (wt: Ø 5.3±0.05 µm, mutant: Ø 5.6±0.12 µm) the variance of R4 axonal length in *gogo* mosaic eyes (between 1.8 to 9.6 µm) was significantly differed from wild-type controls (between 4.3 and 6.5 µm, Levene’s test, p<0.0005, Figure S1). Moreover, the normally uniform circular cartridges of R1–R6 axon termini were deformed and varied in size, reflecting a variant number of axons per target cartridge instead of the normal 6 (Figure 1F’). Because individual R cell types have a stereotyped intrafascicular location, we examined the starting point of individual R4 extensions in *gogo* mutant clones to visualize pre-cartridge selection. We examined the topographic regularity of R4 termini using DeLaunay triangulation and Voronoi diagrams (see material and methods). In wild-type conditions, connecting the starting points of R4 extensions resulted in a net of equal triangles and polygons, reflecting the orderly distribution of pre-cartridges (Figure 1I). However, under mutant conditions, R4 extensions failed to arrange properly leading to a disruption of a uniform retinotopic map clearly visible in the DeLaunay and Voronoi diagrams (Figure 1J). Taken together these results reveal that in the absence of Gogo (i) the well-ordered topographic patterning of R1-6 axon fascicles during ganglion-specific targeting is disrupted and (ii) R1–R6 growth cones fail to select appropriate target cartridges.

### Gogo Function in R8 is Sufficient for Formation of a Smooth Topographic Map at the Lamina Plexus

**Figure 2:**
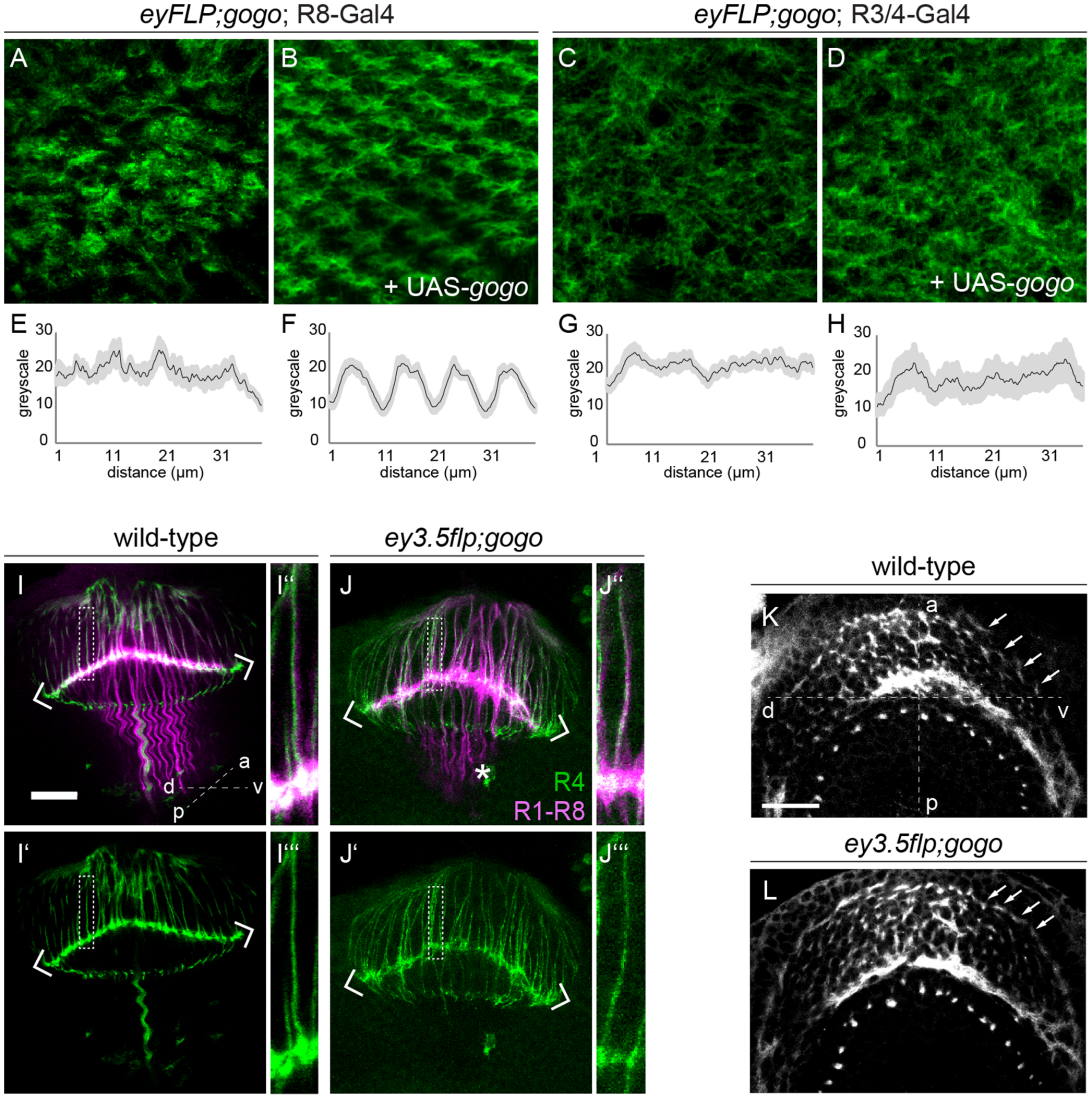
Gogo function in R8 is sufficient to maintain retinotopic map formation. (A–D) Pupal lamina in *eyflp;gogo* eyes (control) and specific rescue experiments stained with mAb24B10 and plot profiles. (A, C) Absence of Gogo in the majority of R axons and the target area strongly disrupts the overall pattern of the pupal lamina (42 hr APF). (B) When FL Gogo is specifically restored in R8 axons the orderly organized pattern of the topographic map is fully rescued. (C) However, FL Gogo expression in R4 axons in the mutant background cannot restore the orderly arrangement of cartridges. (E–H) Pooled plot profiles of 4 cartridges in each experiment provide an example of the pattern regularity. The R8 rescue displays a periodic profile while in mutant controls and R4 rescue a periodicity is not detectable. (I-J’’’) Larval optic lobes in control and *gogo* mosaic animals and corresponding magnifications. mAb24B10 staining visualizes R1–R8 axons (magenta) and GFP is expressed in the R4 subtype (green). While *gogo* mutant axons strongly bundle within the medulla (asterisk), mutant R cell targeting to the lamina plexus (defined by chevrons) shows only mild irregularities compared to wild-type. Single wild-type (I’’) and *gogo* mutant (J’’) R4 axons strictly remain within their fascicle. (K, L) In *eyflp;gogo* eyes R fascicle (white arrows) distribute along the anterior-posterior axis indistinguishable from wild-type. a: anterior; p: posterior; d: dorsal; v: ventral. Scale bars: 20 µm.

Next, we sought to understand the cellular requirement of Gogo function. Earlier data revealed that in third instar larval stages Gogo localizes to the tips of R8 axons only [18]. At 24 hr APF, Gogo expression is detectable also in R1–R6 axons. We therefore tested the possibility that Gogo expression in R8 could be sufficient for topographic mapping. Interestingly, it was proposed that R8 acts as a pioneer axon for the ommatidial fascicle such that in each ommatidium the R8 cell extends first towards the brain, followed by sequential outgrowth of R2/R5, R3/R4, R1/R6 and lastly R7 (Figure 1A [29]). We expressed full length *gogo* cDNA specifically in R8 axons using the specific driver line 109-68-Gal4 in a *eyflp;gogo* background. In *eyflp;gogo* flies the majority of R cells and a small fraction of brain cells are homozygous mutant [20]. 109-68-Gal4 drives expression exclusively in R8 axons but not in target cells surrounding the lamina plexus (Figure S2). Targeted expression of FL-Gogo in R8 (n = 13) rescued the *gogo* mutant phenotype fully compared to controls (n = 8, Figure 2A, 2B, 2E, 2F). The cartridge pattern was indistinguishable from wild-type arguing that not only initial topography but also target selection was rescued. In contrast, when we did the same experiment using the *mδ*-Gal4 driver to drive Gogo expression exclusively in R3/R4 cells, we did not detect a comparable rescue (Figure 2C, 2D, 2G, 2H). *mδ*-Gal4 expresses initially in R3 and R4 and is later confined to R4 only. Therefore, we conclude that Gogo expression in R8 is fully sufficient for topographic map formation. We cannot exclude that re-expression of Gogo in two receptor types simultaneously interferes with efficient rescue and proper axon targeting.

How does Gogo in R8 axons mediate fascicle order along the lamina plexus? We considered the possibility that consistent with a requirement of Gogo for mediating repulsive interactions between R8 axons during medulla targeting, defects in lamina projection could reflect R8 separation defects before entering the medulla. *gogo* mutant growth cones appear more irregular in the developing larval optic lobes (n_wild-type_ = 13, n_mutant_ = 19, Figure 2 I-J’’’). However, a dorsoventral view of larval brains revealed that R axon bundles appear separated from each other at the lamina plexus and do not seem to clump to a similar extend as *fmi* mutant axons ([6], Figure 2K, 2L). Thus, R1–R6 fascicles could be abnormally positioned at the lamina plexus due to either loss of repulsion between R8 axons or loss of axon-target interactions. This could be the case, if Gogo interacted with a so far unknown ligand in the target cells.

### Gogo is not Required in Single R Cells for Cartridge Selection

**Figure 3:**
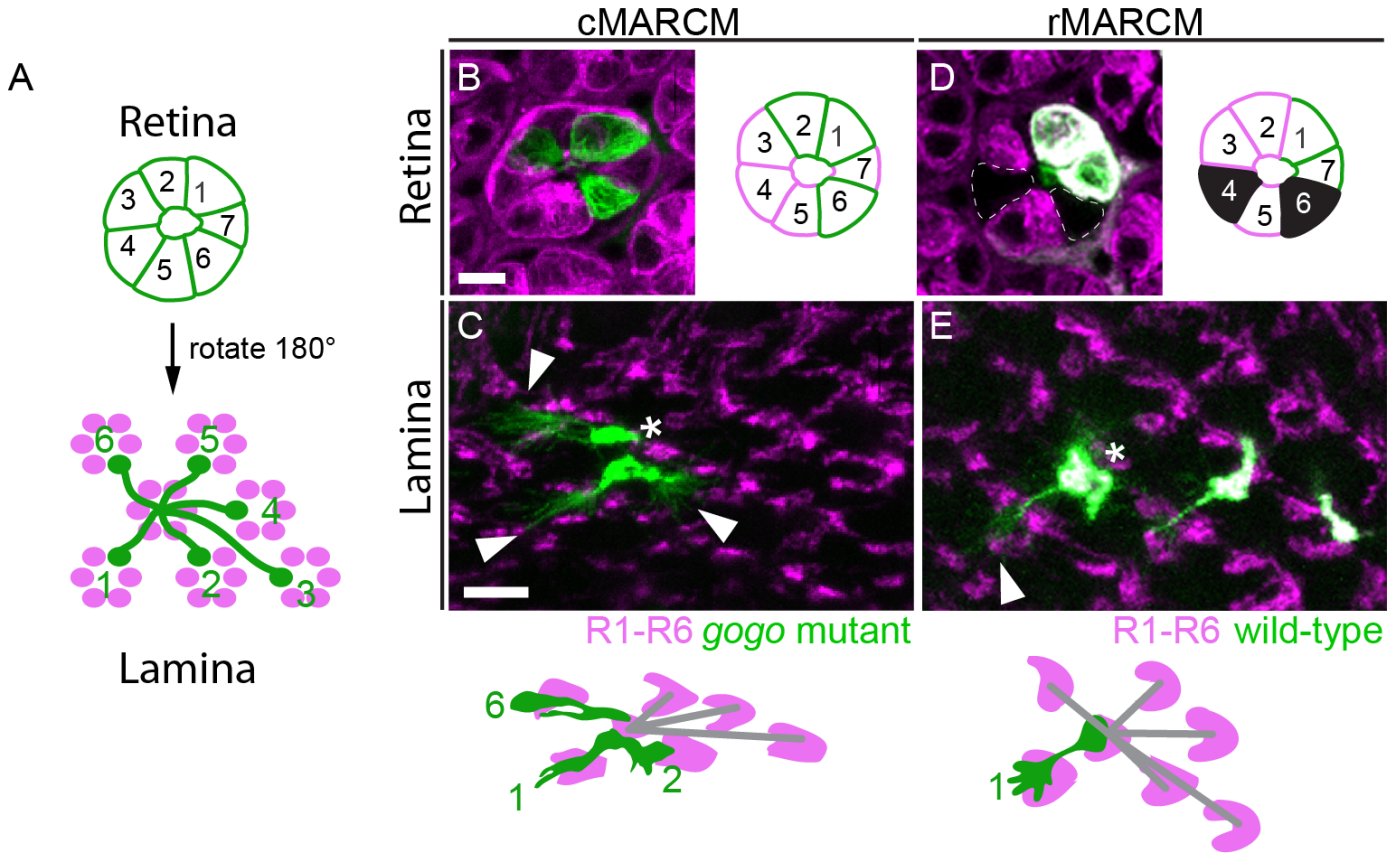
Absence of Gogo in single R cells does not influence cartridge selection. (A) Schematic of cartridge target selection. R1–R6 projection pattern is specific and uniform for each R1–R6 fascicle. Axon extensions occur in the control 180° rotated with respect to the position of their cell bodies. (B–E) Single cells were made homozygous for *gogo* null allele via mitotic recombination. Asterisks mark start and arrowheads end of axon extensions. (B, C) In the complementary MARCM (cMARCM) approach mutant R axons are labeled by mCD8-GFP (green) and wild-type cells are labeled with Gmr-KO (magenta). *gogo* mutant R axons surrounded by wild-type cells choose correct targets in the lamina (C) with respect to the position of their ommatidial cell bodies (B). (D, E) In the reverse MARCM approach (rMARCM) single wild-type axons are labeled with mCD8-GFP in a wild-type Gmr-KO labeled background (magenta) and unlabeled *gogo* mutant R cells that appear in black (dashed lines). Wild-type axons adjacent to mutant axons target correctly (E) with respect to their cell-bodies (D). Scale bars: 5 µm.

Although Gogo expression in R8 cells fully restored the well-ordered pattern of the lamina at 42 hr APF, we considered the possibility that Gogo might mediate afferent-afferent interactions between R1–R6 axons during target cartridge selection. Thus, we next addressed whether Gogo was required in a cell-autonomous manner in single R axons. To test this, we used mosaic analysis with a repressible cell marker (MARCM) [30], [31] and labeled single *gogo* mutant clones with mCD8-GFP. The surrounding cells were wild-type and labeled with MARCM-independent Gmr-KO (complementary (c) MARCM [18]). Mutant clones were generated in L3 larvae using FLP recombinase under the control of the hs-promotor. Because axonal extensions of R cell types in the lamina are stereotypic with respect to the position of their cell bodies, the subtype and the behavior of individual axon classes can be analyzed (Figure 3A [28]). Mutant axon extensions were traced from the retina to the lamina and their lateral projections followed from the cartridge of origin to the target cartridge. In control conditions, clones of single R cells defasciculated properly and innervated correct cartridges (n = 15, data not shown). Also, all projections made by single *gogo* mutant R axons were indistinguishable from the control experiment (n = 20). In addition to single mutant cells, we analyzed ommatidia with two (n = 13) or more (n = 9) mutant cells per fascicle (Figure 3B, 3C). Remarkably, under these conditions mutant axons behaved as wild-type axons, invariant and specified by their original ommatidium. Thus, Gogo is not required cell-autonomously in R1–R6 axons for target cartridge selection.

To examine if Gogo is required non-cell autonomously in R1–R6 cells by supplying a short-range signal for neighboring cells, we used reverse (r) MARCM [17], [32]. In this approach all wild-type cells expressed Gmr-KO and a subset of wild-type cells was labeled in addition with mCD8-GFP. *gogo* mutant R cells did not express any marker. This method allowed us to trace wild-type axons, which were adjacent to *gogo* mutant axons of the same ommatidium. We analyzed wild-type axons directly neighboring a mutant axon (n = 46), and which have either one (n = 19) or two (n = 9) wild-type cells between themselves and the mutant axon (Figure 3D, 3E). Remarkably, in all conditions analyzed axons innervated their appropriate target. Taken together, removal of Gogo in single cells was insufficient to result in any mistargeting phenotype as seen in large mutant clones. Therefore, defects in cartridge selection likely are a secondary consequence of the disordered initial topographic map.

### Loss of Gogo is Compensated by Targeting of Wild-type Neighboring Fascicles

**Figure 4:**
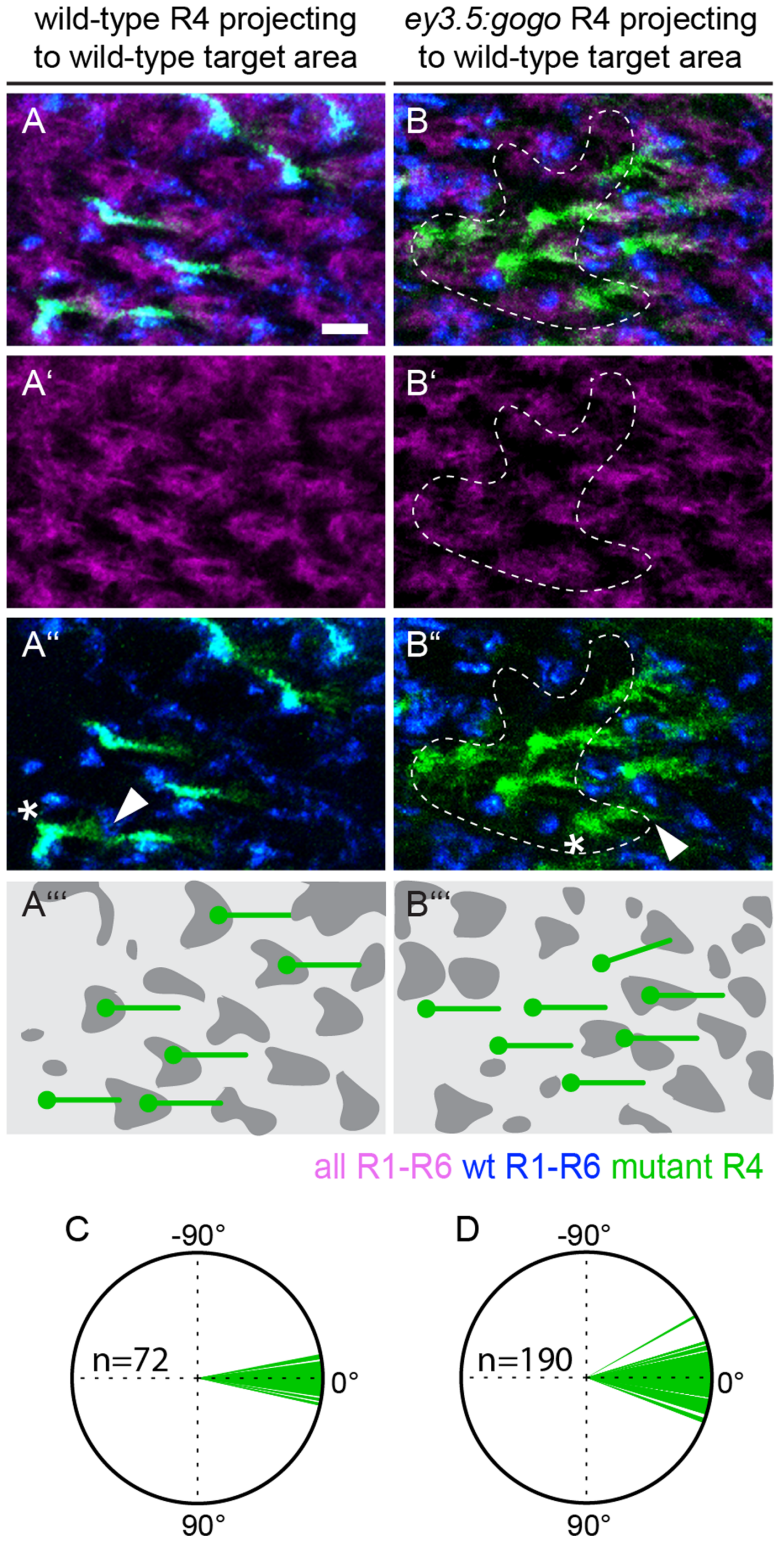
Mutant R4 axons target correctly to areas innervated by wild-type R axons. (A–C’’’) Cartridge pattern and behavior of R4 axons in small *ey3.5flp:gogo* clones and controls. All R1–R6 cells are labeled with mAb24B10 (magenta). Gmr-KO is located in *trans* to the *gogo* mutant allele. Thus, wild-type areas express Gmr-KO (blue) and mutant areas appear black (indicated by dashed lines). mCD8-GFP is expressed under the control of the *mδ-Gal4 driver.* The Gal4 repressor Gal80 is located in *trans* to the control (A–A’’) or the *gogo* mutant allele. Therefore only R4 axons that are homozygous for the control or the *gogo* mutant allele express mCD8-GFP. (A–A’’’) Laminae of control animals display a uniform R4 and a well ordered overall projection pattern. (B–B’’’) Mutant R4 axons express mCD8-GFP, whereas wild-type R4 axons are not visible. The overall pattern of lamina cartridges displays a regular distribution indistinguishable from wild-type. Mutant R4 axons project parallel to each other when R axons of the target cartridge area are wild-type. (D–E) Orientation vectors of control (D) and mutant R4 axons that target to wild-type areas (E). Scale bar: 5 µm.

Our data so far indicated that defects in fascicle ordering when reaching the lamina plexus (i) depend on Gogo function in R8 directly and (ii) that loss of Gogo in R8 cells indirectly influences target cartridge selection of R1-6 axons. In contrast, analysis of *ey3.5flp:gogo* clones (see above) that span only a few neighboring ommatidia (n = 21) demonstrated that fascicle ordering is not altered (5–13 mutant ommatidia surrounded by wild-type area, see material and methods). Thus, the phenotype seen in large clones is not visible (n = 16, between 12 and 15 or more than 15 mutant ommatidia surrounded by wild-type area, Figure 4A–B’’’). We conclude that Gogo has to be absent in a large fraction of neighboring ommatidia to disrupt the order of the retinotopic map. The abnormal phenotype that we observed when Gogo was absent in large areas could therefore reflect a community effect of a large number (>15) of misguided axons. To assess the differences in R1–R6 target cartridge selection between large clones and small clones, we analyzed the behavior of mutant R4 axons that project from the mutant side of the clone border towards the wild-type side (Figure 4 B–B’’’). Indeed, we found that the projection pattern of mutant R4 axons targeting into a wild-type area (n = 190) was indistinguishable from the control experiment (n = 72, Figure 4D, 4E, two-sampled K-S test). We conclude that the more target cartridges become populated with *gogo* mutant R axons and the higher the disorder of the initial lamina topographic map, the larger the influence of Gogo on target cartridge selection.

### Gogo is Crucial for Cartridge Elongation of R1–R6 Axons

Gogo function mediates cartridge formation by instructing distribution of R1–R6 fascicles along the lamina plexus. However, although expressing Gogo exclusively in R8 in a *eyflp;gogo* background rescued initial topography and target cartridge selection, we demonstrated that R8-specific expression of Gogo is not sufficient to fully rescue cartridge assembly, and adult brains still show the *gogo* mutant phenotype [19]. This indicates that Gogo is not only required for the initial topographic map but also during a later step. Therefore, we next assessed the behavior of mutant axons during cartridge elongation.

**Figure 5:**
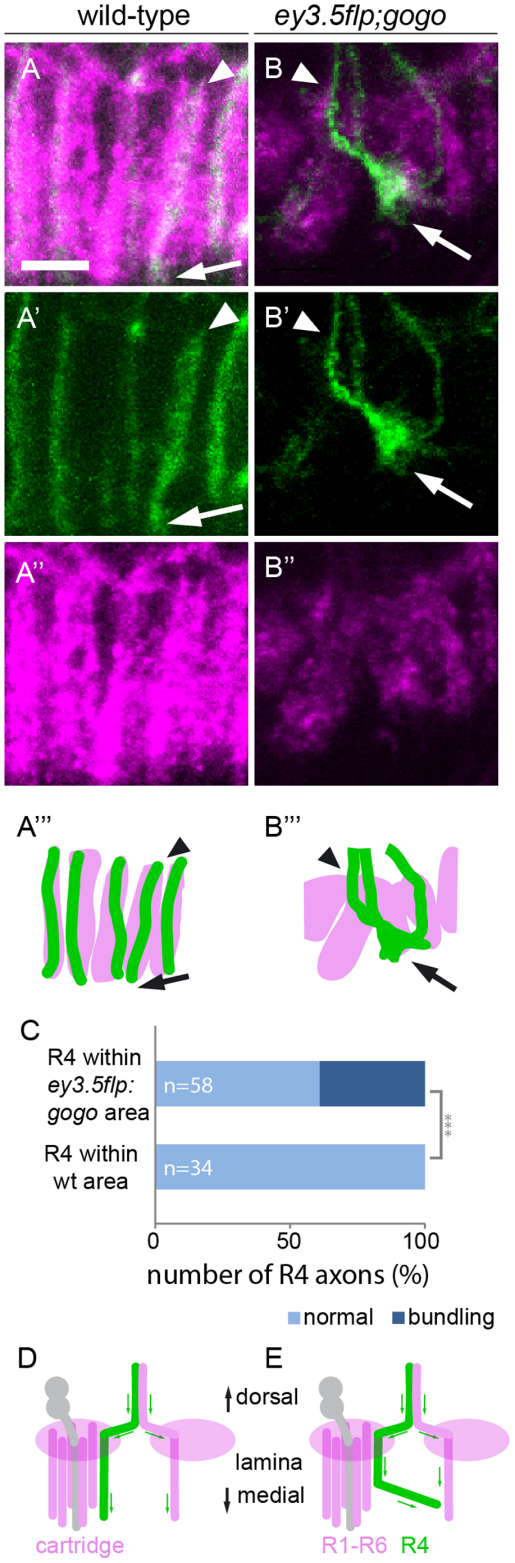
In*gogo* mosaic eyes R axons bundle with R axons from neighboring cartridges. (A–B’’’) Confocal sections showing the developing lamina in wild-type and *gogo*- mosaic eyes and corresponding schematics at 51 hr APF. R1–R6 axons are labeled with Gmr-KO (magenta) and R4 axons are labeled with mCD8-GFP (green). Arrowheads indicate the start and arrows the end of axon elongation within the cartridge. (A–A’’’) In wild-type controls R axons elongate parallel in separate columns. (B–B’’’) When Gogo is absent in the majority of ommatidia R axons fail to project in a parallel fashion. Moreover, single R axons (R4) leave their original target cartridge and bundle with axons of adjacent cartridges. Note that axon termini are not yet fully extended at this developmental stage. (C) In the wild-type control all R4 axons follow the original tracts of their target cartridge. In the *ey3.5flp:gogo* background almost half of R4 axons project away from their target cartridge to join a neighboring column. (D–E) Model of R1–R6 axon extensions during cartridge formation in wild-type (D) and mutant (E) backgrounds. When axons arrive at the lamina plexus they extend lateral to the target cartridges, turn and elongate along lamina neurons (grey). Scale bars: 5 µm.

**Figure 6:**
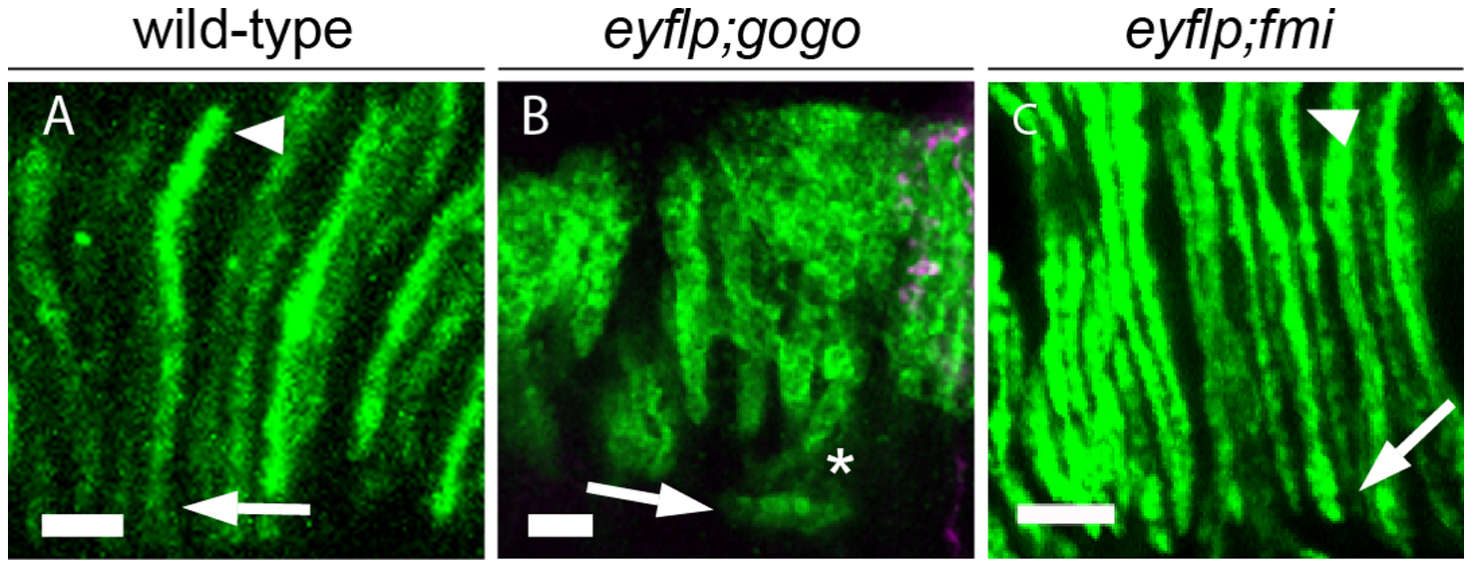
*gogo* mutant but not *fmi* mutant R1–R6 axons bundle within cartridges. (A–C) Agarose sections of adult fly heads showing horizontal view of adult cartridges in wild-type controls, *eyflp;gogo* and *eyflp;fmi* mosaic eyes. Flies carry the Rh1- τlacZ reporter and are stained with anti-β-galactosidase antibody to visualize R1–R6 axons. Arrowheads mark the start (apical) and arrows the end (proximal) of R1–R6 axon extensions. In adult wild-type controls R1–R6 projections are parallel. While in the absence of Gogo R1–R6 axons display a strong bundling (asterisk) within lamina cartridges, the lack of Fmi is indistinguishable from wild-type control. Scale bars: 5 µm.

Upon reaching their final target cartridges at 42 hr APF wild-type R1–R6 axons turn 90° and project proximally to assemble the lamina neuropile [14]. R1–R6 growth cones extend in parallel to neighboring axons and remain tightly associated with axons of their own target cartridge. We analyzed the proximal extensions of R1–R6 axons within the developing lamina in the absence of Gogo. We characterized R4 extensions within cartridges at 51 hr APF. Axons of individual cartridges can be clearly distinguished from neighbors at this developmental stage (Figure 5A–A’’’). As expected, we always found exactly one R4 axon within each cartridge projecting parallel to neighboring R4 axons, straight towards the brain and no change in their position within the cartridges (n = 34). In contrast in *ey3.5flp;gogo*, R4 did not extend straight but turned laterally (n = 58, Figure 5B–B’’’). The staining of all R axons revealed that cartridge bundles project away from their appropriate path. Surprisingly, we found that single R4 cells often do not remain in their target cartridge but project to neighboring cartridges and follow inappropriate tracts (Figure 5C, chi-test, p<0.0001). The observed phenotype within the lamina plexus posed the question whether an inappropriate number of axons per cartridge caused bundling within the lamina plexus. We found evidence that the bundling of R1–R6 termini during proximal axonal extension is due to a primary function of Gogo: It has been demonstrated that in the absence of the atypical cadherin Fmi, axons choose inappropriate targets and thus cause a strong hypo- and hyperinnervation of cartridges [6], [17]. In a lateral view of the adult lamina cartridges contain a variable number of axon termini [6]. Using Rh1-τlacZ to mark R1–R6 termini [20], we analyzed proximal axon projections in adult flies mutant for the *fmi* null allele *fmi^E59^* (n = 26, *eyflp;fmi*). Unlike in the absence of Gogo (n = 15), the extensions of *fmi* mutant axons along cartridge trajectories were indistinguishable from wild-type (n = 9, Figure 6A–C).

**Figure 7:**
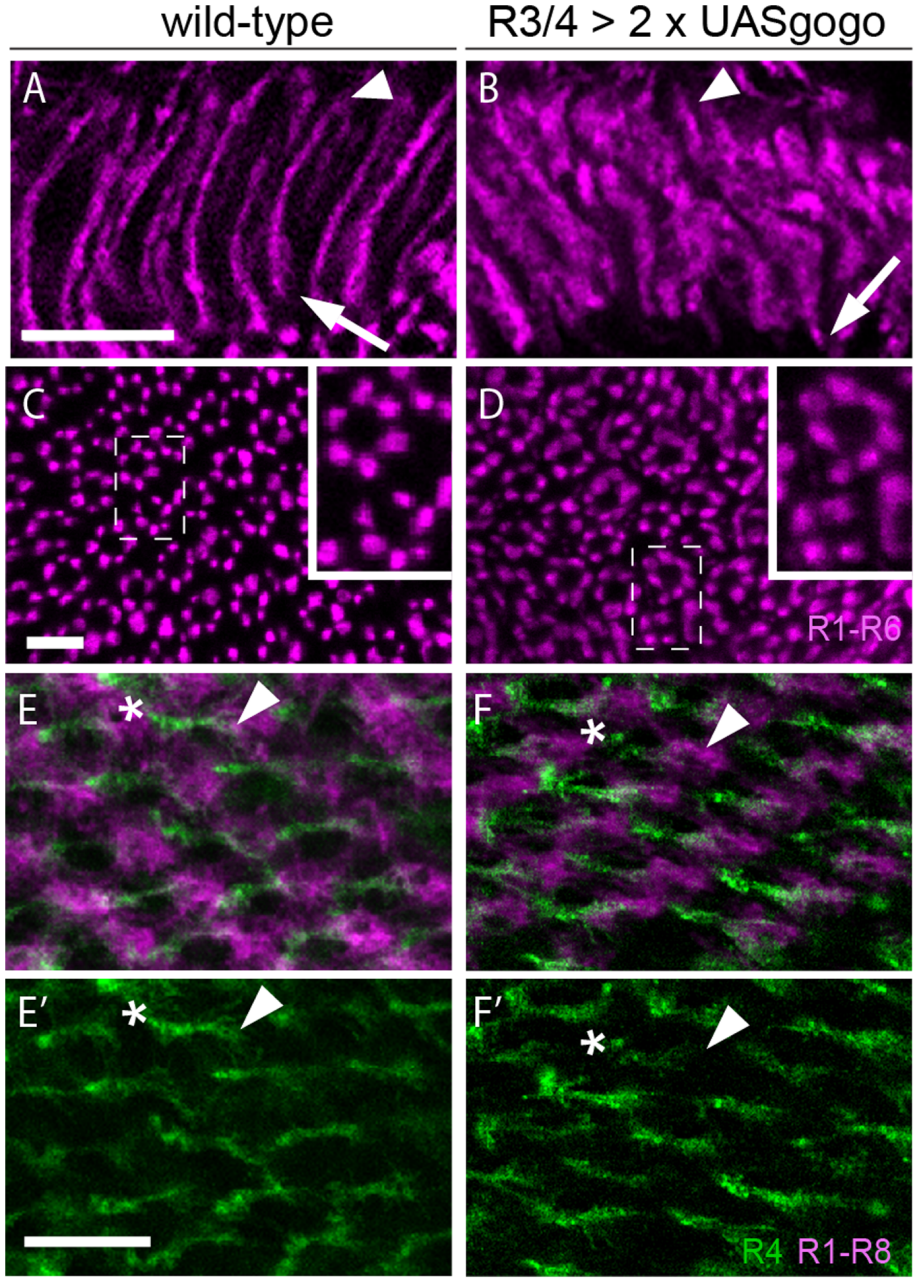
Overexpression in single R cell types disrupts cartridge formation. (A–F) Confocal stacks of wild-type and Gogo overexpression in R3/R4 using the *mδ*–Gal4 driver. (A–D) Horizontal (A,B) and lateral (C, D) patterns in adult brains. Wild-type control in (A, C) and overexpression of Gogo in (B, D). Termini of R1–R6 axons are visualized using the directly fused construct Rh1-lacZ. (A, B) Compared to wild-type (A), the pattern of R1–R6 axons in gain of function flies is disrupted (B). Arrowheads mark the start points and arrows the end of R4 extensions. In wild-type (C) cartridges are formed by six axon termini (rings). The number of termini per cartridge is altered when Gogo levels are increased in R3/R4 (D). (E, F’) Wild-type control (E, E’) and overexpression (F, F’) in pupal laminae. Asterisks mark the start points and arrowheads the end of R4 extensions. R1–R6 cells are labeled with mAB24B10 antibody staining and R4 axons are visualized by mCD8-GFP expression. Increasing Gogo levels in R3/R4 axons does not influence R4 target selection or the overall pattern of cartridge assembly. Scale bars: A–B’, E–F = 5 µm, C, D: 10 µm.

We next analyzed Gogo overexpression in the adult lamina. Using Rh1-τlacZ [20], we found that overexpressing Gogo in R3/R4 neurons caused similar bundling defects in cartridge elongation as observed in mutants (Figure 7A, 7B). We also analyzed the lateral pattern of cartridge assembly in the gain of function background and observed that 55% of all cartridges (n = 256) contain abnormal numbers of R1–R6 axons, compared to only 3% abnormal cartridge numbers in the wild-type control (n = 375, Figure 7C, 7D). To address whether putative bundling of R1-6 axons during earlier stages affects cartridge selection, we overexpressed two copies of full-length Gogo using the *mδ*-Gal4 driver. In pupal stages at the onset of target cartridge selection (42 hr APF) the increase of Gogo levels in R3/R4 neurons (n = 16) did not disrupt R4 target selection or the overall pattern of cartridges (Figure 7E–F’). This result was consistent with our finding that Gogo does not mediate repulsion among R1–R6 axons (Figure 3). Thus, while hypo- and hyperinnervation of cartridges during cartridge selection at the lamina plexus is independent of Gogo-mediated R1-R6-axon interaction, Gogo-dependent prevention of bundling at the level of cartridge elongation influences cartridge innervation also at very late stages during eye development (Figure 5D, 5E).

## Discussion

In this study we elucidate the mechanisms underlying the remarkable precision of R1–R6 superposition in the fly lamina. Our results indicate that the transmembrane receptor Gogo instructs retinotopic map formation in the lamina at two important developmental time points. Photoreceptor fascicles lacking Gogo function display defects in their spatial distribution along the lamina plexus during early developmental stages. Re-expressing Gogo solely in R8 axons rescues the orderly arrangement of photoreceptor fascicles fully, providing evidence for a role of Gogo in R8 in instructing initial topographic map formation and cartridge innervation of R1–R6 photoreceptor neurons. Pioneer-follower interactions between the first differentiating R8 axon and R1–R6 have been proposed before [6], [33]. The atypical cadherin Fmi is sufficient in R8 for initial topographic mapping by mediating repulsive interactions between R8 axons before exiting the lamina [6]. It is important to note that Fmi and Gogo genetically cooperate in some aspects of R8 layer specificity in the medulla [19]. Like in *fmi* mutants, R axons are abnormally positioned within the lamina plexus in the absence of Gogo. However, irregularities of R1–R8 axons in the lamina in *gogo* mutants seem to be milder compared to *fmi* mutants (Lee et al., 2003). Moreover, the dorsoventral position of R axon fascicles is not altered in *gogo* mutants. Thus, at this developmental stage Gogo could either mediate R8 axon-axon interactions or interactions between R8 axons and target cells in the lamina.

In a second developmental stage, R1–R6 axons fail to select appropriate target cartridges in the absence of Gogo. Several studies revealed that afferent-afferent interactions among R axon growth cones of the same fascicle mediate their specific lateral directions during cartridge selection [6], [9], [17], [27], [31], [34], [35], [36]. However, Gogo is not mediating afferent-afferent interactions between R1–R6 growth cones as loss of Gogo in single R cells does not affect target cartridge selection. Fmi is mediating non-autonomous homophilic interactions to guide R1–R6 axons to target cartridges [17]. Unlike Fmi, we show that Gogo is not required non-autonomously in R1–R6 axons to select proper target cartridges. Thus, Fmi function is clearly independent of Gogo activity during target cartridge selection. How does Gogo function contribute to R1–R6 target specificity?

Although R1–R6 axonal extensions are cell-type specific and asymmetric during cartridge selection, R1–R6 fascicles and lamina neuron targets are identical [16]. They only differ in their anterior-posterior and dorso-ventral position at the lamina plexus. Our results show that the absence of Gogo alters the positional map of axon fascicles. We find that in this case, R4 axons shift in direction and vary significantly in their axonal length. This stresses the importance of proper spacing between axon fascicles for R1–R6 target cartridge selection. Our results complement an earlier study, where diagonal but not mirror-reflecting rotation of ommatidia disrupts R axon extension in respect to the position of its cell body [28].

Interestingly, we found that Gogo is also required in the last developmental step, when R axons elongate within their target cartridges and form synapses [14]: In the absence of Gogo, R axons fail to stay in their appropriate cartridges and bundle with R axons of neighboring cartridges. The defects are similar to those described for R8 during medulla targeting [18], suggesting that Gogo mediates repulsive interactions between neighboring R axons and/or lamina neurons selectively after initial cartridge selection in the lamina. To our knowledge, we describe the first axon guidance phenotype during cartridge elongation. Thus, we provide new insights of how the highly precise connection specificity of lamina cartridge maintenance is achieved during development. Moreover, we show that Fmi is not required for normal extension within the lamina plexus. Therefore, Gogo directly, and not as a secondary effect, regulates cartridge elongation and acts independent of Fmi during cartridge elongation. Although both proteins display the same phenotypes in adult cartridges, their underlying function during cartridge formation is different.

Finally, our data highlights the importance of coordinated interactions of redundant guidance mechanisms: we find that a collapse of the retinotopic map occurs only when Gogo is absent in a group of neighboring neurons but not in single neurons or very small clones. We propose a model in which redundant axon guidance mechanisms compensate for the loss of Gogo in single R axons or very small regions of mutant neighboring fascicles. Interestingly, a study in zebrafish proposed that the phenotype of a guidance molecule can be reduced to an undetectable level by complementing guidance mechanisms [37]. We suggest that guidance by neighboring axons or fascicles narrows the area of choice each fascicle and thereby compensates for the loss of Gogo in single R axons or very small regions of mutant neighboring fascicles. When the molecular label Gogo is missing in only one or a few cells, their degree of freedom is still restricted by the fact that the surrounding axons follow their proper guidance target. Interestingly, computational and *in vivo* models addressed the co-dependence of spatial competition and axon-target interactions in the mouse visual system. The study concluded that chemical labels are insufficient to specify the retinocollicular projection, but instead competition for space is required during map formation [38].

Based on our data, we propose that the extraordinary precision of connectivity in the Drosophila lamina is facilitated by Gogo-dependent guidance by R8 and neighboring axons and fascicles in three different developmental stages. Gogo function can be partially compensated for by the presence of neighboring, correctly targeting wild-type R axons or axon fascicles that provide spatial restriction and guidance within the limited space of the fly retina.

## Supporting Information

Figure S1 **Boxplots of R4 axonal length in wild-type and** ***ey3.5flp;gogo*** **laminae (42 hrs APF).** In wild-type laminae, the length of R4 axons varies between 4.3 and 6.5 µm. In the *ey3.5flp;gogo* background R4 axon length is significant different from wild-type: The length of R4 axons varies between 1.8 and 9.6 µm.
(TIF)

Figure S2 **Expression pattern of the driver Gal4[109-68] in different developmental stages.** UAS-mCD8-GFP is expressed under the control of Gal4[109-68] (green). R1-8 cells are labeled by 24B10 antibody staining (magenta) and neurons with elav antibody staining (blue). Lamina neurons are indicated by chevrons, the lamina plexus is defined by chevrons and arrowheads mark R8 axons in the medulla. In all developmental stages (3^rd^ instar larval, 24 and 48 hr APF) expression of Gal4[109-68] is detected in R8 axon cell bodies in the retina (asterisks). In cells surrounding the developing lamina (including lamina neurons) Gal4[109-68] is not expressed. However, in deeper brain regions (including cells surrounding the developing medulla) expression is widely detectable. Scale bars: 30 µm.
(TIF)
