# Supplementary figures and images for: Gogo Receptor Contributes to Retinotopic Map Formation and Prevents R1-6 Photoreceptor Axon Bundling
Source: PLoS One. 2013 Jun 24;8(6):e66868. doi: 10.1371/journal.pone.0066868 (PMC3691217; doi:10.1371/journal.pone.0066868)

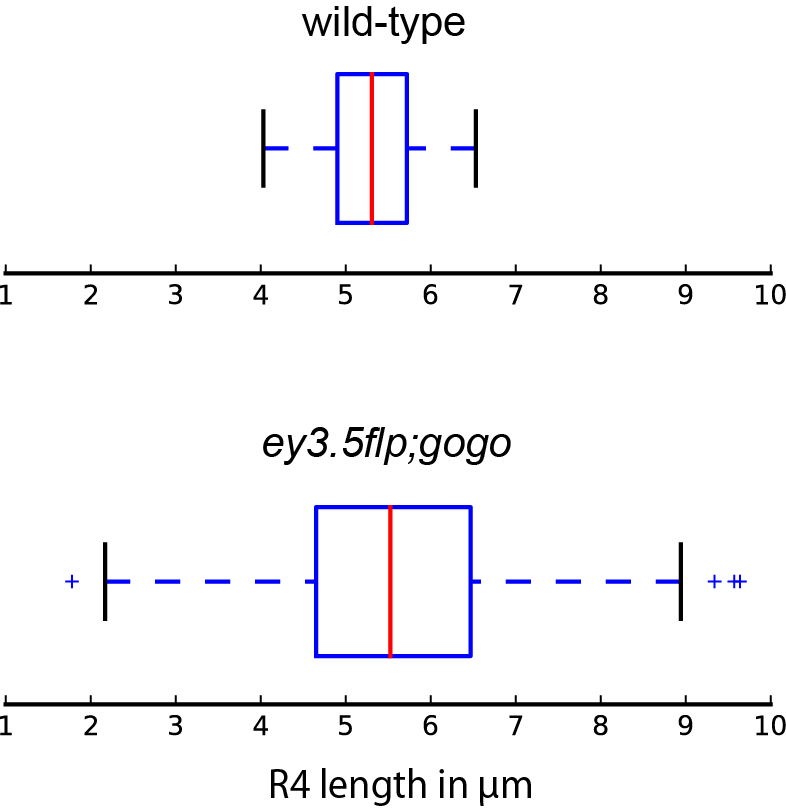

Supplement: Figure S1 — Boxplots of R4 axonal length in wild-type and ey3.5flp;gogo laminae (42 hrs APF). In wild-type laminae, the length of R4 axons varies between 4.3 and 6.5 µm. In the ey3.5flp;gogo background R4 axon length is significant different from wild-type: The length of R4 axons varies between 1.8 and 9.6 µm. (TIF) [file pone.0066868.s001.tif]

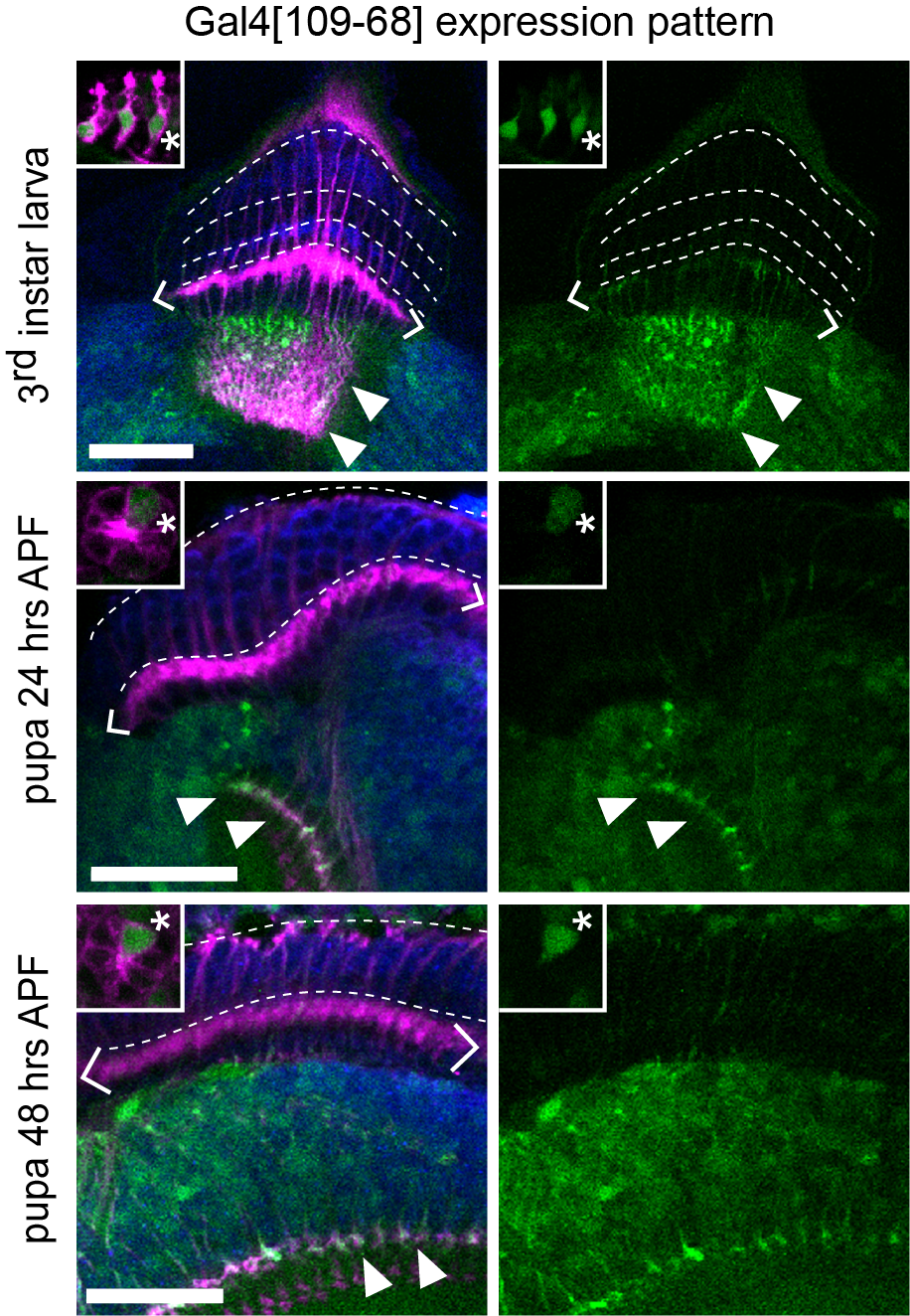

Supplement: Figure S2 — Expression pattern of the driver Gal4[109-68] in different developmental stages. UAS-mCD8-GFP is expressed under the control of Gal4[109-68] (green). R1-8 cells are labeled by 24B10 antibody staining (magenta) and neurons with elav antibody staining (blue). Lamina neurons are indicated by chevrons, the lamina plexus is defined by chevrons and arrowheads mark R8 axons in the medulla. In all developmental stages (3rd instar larval, 24 and 48 hr APF) expression of Gal4[109-68] is detected in R8 axon cell bodies in the retina (asterisks). In cells surrounding the developing lamina (including lamina neurons) Gal4[109-68] is not expressed. However, in deeper brain regions (including cells surrounding the developing medulla) expression is widely detectable. Scale bars: 30 µm. (TIF) [file pone.0066868.s002.tif]
